# Impact of pediatric urgent care clinics on emergency department utilization and patient flow: a pre–post cohort study in Saudi Arabia

**DOI:** 10.1186/s12873-026-01670-0

**Published:** 2026-07-08

**Authors:** Abdulhameed Al Khalaf, Alla Albisher, Ahmed Al Shams, Mohammed Al Khalaf, Jassem Althani, Jassim Al Essa, Ali Al Ali, Mohammed Alwabari, Haidar Alhassan

**Affiliations:** 1 Family Medicine Department, Al-Ahsa, Saudi Arabia; 2 Pediatric Emergency Department, Alahsa Health Cluster, Alahsa, Saudi Arabia; 3 Pediatric Emergency Fellowship Program, Al-Ahsa, Saudi Arabia; 4 Emergency Nursing Services, Alahsa Health Cluster, Alahsa, Saudi Arabia

**Keywords:** Pediatric emergency department, Urgent care, Overcrowding, CTAS, Length of stay, Saudi Arabia

## Abstract

**Background:**

Pediatric emergency departments (PEDs) frequently experience overcrowding, much of it related to low-acuity visits that may be manageable in outpatient settings. Pediatric Urgent Care Clinics (PUCCs) have been introduced to help accommodate such demand, but evidence from the Gulf region remains limited.

**Objectives:**

To evaluate changes in PED utilization, acuity mix, and patient-flow indicators following implementation of a PUCC at the Maternity and Children’s Hospital in Alahsa, Saudi Arabia.

**Methods:**

We conducted a retrospective pre–post observational cohort study using electronic health record data from October 2023 to June 2024 (pre-implementation) and October 2024 to June 2025 (post-implementation). Variables included Canadian Triage and Acuity Scale (CTAS) level, timestamps for triage, physician start, and discharge, and demographic characteristics. Outcomes included PED visit volume, acuity distribution, door-to-doctor time, and length of stay (LOS). Continuous variables were summarized using medians with interquartile ranges (IQRs) and 5% trimmed means; categorical variables were compared using chi-square tests, and effect sizes for binary outcomes were expressed as risk ratios (RRs) with 95% confidence intervals.

**Results:**

A total of 147,175 PED encounters were analyzed, including 84,595 in the pre-implementation period and 62,580 in the post-implementation period. In the post-implementation period, an additional 24,962 encounters were managed in the PUCC. The proportion of low-acuity PED visits (CTAS 4–5) decreased from 81.1% to 50.2% (RR 0.62; 95% CI 0.61–0.62; *p* < 0.001). Mean daily PED encounter volume declined from 308.7 to 229.2 encounters/day (incidence rate ratio 0.74; 95% CI 0.74–0.75; *p* < 0.001). Median door-to-doctor time was 37 min (IQR 22–63) before implementation and 39 min (IQR 22–65) after implementation, while median LOS increased from 62 min (IQR 34–112) to 76 min (IQR 43–131) (*p* < 0.001 for distributional comparisons).

**Conclusion:**

Implementation of a PUCC was associated with lower PED visit volume, a reduced proportion of low-acuity PED encounters, and a small absolute difference in door-to-doctor time, while PED LOS was longer in the post-implementation period. These findings suggest that a PUCC model may help redistribute lower-acuity pediatric encounters within a hospital-based urgent and emergency care system. Further studies are needed to evaluate safety outcomes and to better account for operational confounding.

**Supplementary Information:**

The online version contains supplementary material available at https://doi.org/10.1186/s12873-026-01670-0.

## Introduction

Pediatric emergency departments (PEDs) are essential for the management of children with serious or potentially life-threatening conditions. However, increasing demand, particularly from low-acuity presentations, has contributed to persistent overcrowding in many settings. Overcrowding is associated with delays in care, increased staff burden, and strain on healthcare systems, especially when emergency services are used for conditions that could be managed in outpatient settings [1]. Prior studies have shown that PED crowding is linked to treatment delays, reduced quality of care, higher admission rates, and more frequent return visits, particularly among children with chronic illnesses such as asthma or sickle cell disease [2, 3]. Despite ongoing efforts to improve triage and patient flow, the mismatch between demand and system capacity remains a major concern internationally [4].

Pediatric urgent care clinics (PUCCs) are intended to manage common acute conditions, such as fever, upper respiratory symptoms, mild gastrointestinal illness, and minor injuries, in children who do not appear to require full emergency-level care [5, 6]. Compared with general urgent care centers, PUCCs are designed around pediatric needs, including child-focused environments, age-appropriate equipment, and referral pathways for escalation when necessary [7]. Utilization of urgent care services by children has increased in other health systems [8], and some reports suggest that these services may reduce non-emergent ED use during operational hours [9]. However, much of the existing literature has focused on general urgent care models or adult populations, and pediatric-specific evidence remains limited [7].

The Canadian Triage and Acuity Scale (CTAS) is widely used to classify emergency presentations according to urgency, from Level 1 (resuscitation) to Level 5 (non-urgent) [10]. In pediatric practice, a substantial proportion of visits fall within CTAS levels 4 and 5, representing children with lower-acuity conditions that may be safely managed outside the emergency department [11, 12]. Worldwide, non-urgent pediatric ED visits account for an important share of overall activity, with reported proportions ranging from 15% to 60% and reaching approximately 60% in some Asian settings [13, 14]. These patterns highlight the pressure placed on PEDs by low-acuity activity and support the need for alternative care pathways [15, 16].

Similar patterns have been reported in Saudi Arabia and the Gulf region. In one Saudi study, more than half of pediatric emergency visits were classified as CTAS 4–5 [17]. Regional reports suggest that this pattern is driven by multiple factors, including caregiver preference for rapid access, limited after-hours pediatric services, and the perception that emergency care offers more comprehensive evaluation [17, 18]. Together, these findings support the development of structured alternatives for children with lower-acuity acute complaints.

To date, published evidence from Saudi Arabia and the Gulf region on the impact of pediatric-specific urgent care models remains scarce. Evaluating the implementation of a PUCC in this context may help clarify whether such services are associated with changes in PED visit volume, acuity mix, and patient-flow indicators in routine practice.

The main objective was to evaluate whether implementation of a protocol-directed, hospital-based Pediatric Urgent Care Clinic was associated with redistribution of pediatric acute-care encounters between the pediatric emergency department and the PUCC. Specifically, we assessed changes in PED encounter volume, acuity mix, door-to-doctor time, and length of stay before and after PUCC implementation. PUCC encounters during the post-implementation period were summarized descriptively to contextualize total pediatric acute-care activity and to avoid interpreting changes in PED volume as changes in underlying demand.

## Materials and methods

### Study design

We conducted a retrospective pre–post observational cohort study using routinely collected electronic health record (EHR) data to evaluate changes in pediatric emergency department (PED) utilization and patient-flow metrics after implementation of a Pediatric Urgent Care Clinic (PUCC) service at Maternity and Children’s Hospital (MCH), Al-Ahsa, Kingdom of Saudi Arabia. To reduce the influence of seasonality, two a priori nine-month observation windows were selected: October 1, 2023, to June 30, 2024 (pre-implementation period), and October 1, 2024, to June 30, 2025 (post-implementation period).

The primary analytic comparison was between PED encounters during the pre-implementation period (PED Pre) and PED encounters during the post-implementation period (PED Post). In addition, encounters managed in the PUCC during the post-implementation period were identified using clinic or location codes and summarized descriptively to characterize the activity and acuity profile of the new service. PUCC encounters were not treated as a post-intervention PED cohort and were not included in the primary pre–post PED comparisons. However, PUCC encounters were summarized together with post-implementation PED encounters in descriptive analyses to contextualize overall pediatric acute-care activity after implementation.

### Setting and service model

The study was conducted at Maternity and Children’s Hospital (MCH), a pediatric referral hospital in Al-Ahsa, Saudi Arabia, and included encounters managed in the pediatric emergency department (PED) and, after implementation, in the Pediatric Urgent Care Clinic (PUCC) service. Pediatric status followed the hospital’s operational age definition and was applied uniformly through routine registration and triage processes.

The PUCC was introduced as a hospital-based service intended to manage lower-acuity acute pediatric presentations that did not appear to require full emergency-level care. The PED and PUCC were located within the same hospital campus. The PUCC operated during defined service hours, while the PED remained open 24 h per day, 7 days per week. Patients were managed according to the institutional triage and eligibility pathway. In general, clinically stable children with lower-acuity presentations were eligible for PUCC management, whereas clinically unstable children and those judged to require emergency evaluation, resuscitative resources, advanced monitoring, or immediate specialist intervention continued to be managed in the PED. Allocation to the PUCC was therefore protocol-directed rather than analyzed as unrestricted family choice between the PED and PUCC.

Both services operated within the same public hospital system. Encounters in the electronic health record were classified as PED or PUCC using site-specific clinic or location identifiers. Because this was a real-world service evaluation rather than a randomized allocation model, the possibility of selection effects and case-mix differences between periods was considered in the interpretation of findings. Patient-level data on eligibility decisions, family preference, refusal of PUCC care, or reasons for remaining in the PED despite low-acuity triage were not available in the extracted dataset.

Analyses were based on de-identified EHR extracts generated by the hospital data team using standardized queries and transferred under institutional data-use agreements.

### Institutional triage and PUCC eligibility pathway

All unscheduled pediatric presentations were assessed through the institutional triage pathway. The PED remained the 24-hour emergency service for children requiring emergency-level assessment, resuscitation, advanced monitoring, urgent specialist input, or management of potentially unstable conditions. Children with CTAS 1–2 presentations, clinically unstable children, and children requiring immediate emergency resources remained in the PED.

The PUCC functioned during defined service hours as a protocol-directed pathway for clinically stable children with lower-acuity acute presentations. Eligible presentations generally included common acute pediatric complaints not requiring resuscitative or advanced emergency resources, such as fever without instability, upper respiratory symptoms, mild gastrointestinal illness, minor injuries, and other stable low-acuity complaints. CTAS 4–5 encounters were considered the main target population for PUCC management, while selected clinically stable CTAS 3 encounters could be managed in the PUCC if they did not require emergency-level monitoring or immediate intervention.

Patients whose condition exceeded PUCC capacity, deteriorated during evaluation, or required escalation were redirected or transferred to the PED according to the institutional pathway. A structured summary of the institutional PUCC triage and eligibility pathway is provided in Supplementary Table 1.

### Participants: inclusion and exclusion criteria

Eligible encounters included all unscheduled pediatric presentations occurring during the study windows and managed in either the PED or, during the post-implementation period, the PUCC, regardless of final disposition, including discharge, admission, transfer, leaving without being seen (LWBS), or leaving against medical advice (LAMA).

Excluded encounters were scheduled or elective visits, direct inpatient admissions without PED or PUCC triage or physician assessment, non-clinical or test registrations, duplicate records, encounters outside MCH, and records with impossible temporal sequences after data-quality checks.

For time-based outcomes, encounters missing the required timestamps after plausibility screening were excluded only from the corresponding time-based analysis and retained for all other eligible analyses. For acuity analyses, CTAS values were restricted to valid levels 1 through 5; encounters coded as 0 or missing were retained in total encounter counts but excluded from CTAS-stratified analyses.

### Data sources, variables, and outcomes

Data extracts included administrative timestamps, including registration, triage, physician start, disposition order, and discharge; clinical variables, including Canadian Triage and Acuity Scale (CTAS) level, chief complaint, and disposition category; demographic characteristics, including age, sex, and nationality; and site identifiers distinguishing PED from PUCC encounters. All datasets were de-identified before analysis, with direct identifiers removed and free-text fields screened to minimize the risk of inadvertent disclosure.

The primary outcomes for the PED pre–post comparison were:

1. PED acuity distribution, with low acuity defined a priori as CTAS levels 4–5 among encounters with valid CTAS 1–5 coding.
2. Door-to-doctor time, defined as the interval from triage to physician start.
3. Length of stay (LOS), defined as the interval from triage to discharge.

The secondary outcomes for the PED pre–post comparison were daily and weekly PED encounter volumes overall and by acuity stratum, defined as CTAS 1–3 versus CTAS 4–5.

Because disposition taxonomies differed between periods, admission outcomes were considered exploratory and interpreted cautiously. They were not treated as a principal effectiveness endpoint. Descriptive PUCC outcomes during the post-implementation period included total encounter volume, CTAS distribution, and disposition categories. These analyses were intended to characterize the case mix managed in the new service and were not used as formal inferential comparisons with PED cohorts.

### Data management and quality control

Dates and times were parsed using day-first formatting and combined into ISO 8601 date-time fields. Negative intervals were considered invalid for time-based analyses and excluded from those analyses, while flagged records were retained in the master dataset for auditability.

Prespecified plausibility thresholds were applied to reduce the influence of clearly implausible time values: 0 to 360 min for door-to-doctor time, 0 to 720 min for doctor-to-decision time, and 0 to 1,440 min for LOS. The doctor-to-decision interval was examined only descriptively and was not included among the primary outcomes. Values falling outside the prespecified ranges were excluded from the relevant primary time-based analyses. CTAS values outside levels 1 to 5 were treated as missing for stratified analyses.

Data-cleaning rules and variable dictionaries were maintained under version control before final analysis.

### Statistical analysis

All analyses were two-sided, and statistical significance was defined as *p* < 0.05. Reporting was guided by the STROBE statement for observational studies.

Continuous variables were summarized as medians with interquartile ranges (IQRs) and, as a robustness measure, 5% trimmed means. The primary statistical comparison was between PED Pre and PED Post cohorts. Distributions of continuous outcomes were compared using the Mann–Whitney U test, with Hodges–Lehmann median differences and 95% confidence intervals reported where applicable. Welch’s t-test applied to trimmed means was used as a sensitivity check for mean-based comparisons.

Categorical variables were summarized as counts and percentages and compared using chi-square tests or Fisher’s exact test when appropriate. Effect sizes for binary outcomes were expressed as risk ratios (RRs) with 95% confidence intervals.

Daily PED encounter rates were compared descriptively across study periods, and incidence rate ratios (IRRs) with 95% confidence intervals were calculated for overall daily volume. Sensitivity analyses examined the influence of alternative handling of missing triage timestamps, using registration time as a proxy where appropriate, and the effect of outlier exclusion rules on time-based outcomes.

PUCC encounters were summarized descriptively using the same definitions but were not included in the formal pre–post hypothesis-testing framework. Given the observational before–after design, the analyses were intended to estimate associations rather than establish causality.

### Sample size and ethics

All eligible encounters during the study periods were included, and no formal sample size calculation was performed. Given the large operational dataset, the study was expected to provide precise estimates for the primary outcomes.

The study used retrospectively collected, de-identified EHR data and involved no direct patient contact. Institutional Review Board approval was obtained from the Maternity and Children’s Hospital, Alahsa (IRB No. MCH-PED-2024-047), which granted a waiver of informed consent in accordance with applicable institutional and national regulations. Data were stored on secure hospital servers with restricted access. Statistical analyses were conducted using R (version 4.x) and Python (version 3.11).

## Results

A total of 84,595 PED encounters were recorded during the pre-implementation period (1 October 2023 to 30 June 2024) and 62,580 PED encounters during the post-implementation period (1 October 2024 to 30 June 2025). In the post-implementation period, an additional 24,962 encounters were managed in the PUCC.

When PED and PUCC encounters were considered together, total post-implementation pediatric acute-care activity comprised 87,542 encounters, including 62,580 PED encounters and 24,962 PUCC encounters. This combined post-implementation volume was higher than the pre-implementation PED volume of 84,595 encounters. Therefore, the observed decrease in PED volume should be interpreted as redistribution of encounters across care settings rather than as a reduction in overall pediatric acute-care demand.

Key timestamp completeness improved in the post-implementation PED cohort. Triage time was recorded for 69,583 of 84,595 encounters (82.3%) in the pre-implementation period and 57,439 of 62,580 encounters (91.8%) in the post-implementation period. Doctor-start time was available for 72,184 encounters (85.3%) pre-implementation and 58,796 encounters (94.0%) post-implementation. Discharge time was available for all encounters in both periods. Valid CTAS levels (1–5) were recorded in 77,498 encounters (91.6%) pre-implementation and 60,737 encounters (97.1%) post-implementation **(**Table 1**)**.

**Table 1:** Cohort size and data completeness by period

Metric	PED Pre	PED Post	*P*-value
Total encounters, n	84,595	62,580	
Triage time, n (%)	69,583 (82.3)	57,439 (91.8)	< 0.001
Doctor-start, n (%)	72,184 (85.3)	58,796 (94.0)	< 0.001
Discharge time, n (%)	84,595 (100)	62,580 (100)	NA
Valid CTAS 1–5, n (%)	77,498 (91.6)	60,737 (97.1)	< 0.001

“PED Pre” = pediatric emergency department encounters from 1 Oct 2023–30 Jun 2024 (pre-implementation). “PED Post” = pediatric emergency department encounters from 1 Oct 2024–30 Jun 2025 (post-implementation). CTAS = Canadian Triage and Acuity Scale; valid levels are 1–5. P-values were derived from chi-square tests comparing proportions between periods; p-value for discharge time was not applicable because completeness was 100% in both groups

Patient demographics and visit characteristics for PED encounters are summarized in Table 2. The median age decreased from 4.67 years in the pre-implementation period to 3.42 years in the post-implementation period. The proportion of male patients was similar across periods (56.5% vs. 55.8%). Most PED encounters occurred on weekdays in both periods (66.9% pre-implementation vs. 68.5% post-implementation). With respect to arrival time, daytime presentations accounted for 29.7% of encounters pre-implementation and 25.4% post-implementation, evening presentations for 42.4% and 38.8%, respectively, and night-time presentations for 27.9% and 35.8%, respectively.

**Table 2:** Demographic and visit characteristics of PED encounters

Characteristic	PED Pre	PED Post	*P*-value
Age, median (years)	4.67	3.42	
Male, %	56.5	55.8	0.008
Weekday arrivals, %	66.9	68.5	< 0.001
Weekend arrivals, %	33.1	31.5	< 0.001
Daytime, %	29.7	25.4	< 0.001
Evening, %	42.4	38.8	< 0.001
Night, %	27.9	35.8	< 0.001

Weekday = Monday–Friday; Weekend = Saturday–Sunday. Time blocks: Daytime 08:00–15:59, Evening 16:00–23:59, Night 00:00–07:59. “Pre” = 1 Oct 2023–30 Jun 2024 (pre‑implementation); “Post” = 1 Oct 2024–30 Jun 2025 (post‑implementation). P-values for categorical variables were derived using chi-square tests

Mean daily PED encounter volume was lower in the post-implementation period than in the pre-implementation period. Across 274 observed days pre-implementation, the mean daily volume was 308.7 encounters/day, compared with 229.2 encounters/day across 273 observed days post-implementation. The incidence rate ratio for daily PED encounters was 0.74 (95% CI 0.74–0.75; *p* < 0.001) **(**Table 3**).**

**Table 3:** Daily pediatric emergency department encounter rates

Metric	PED Pre	PED Post	Incidence Rate Ratio (IRR)	95% CI	*P*-value
Observed days, n	274	273	0.74	0.74–0.75	< 0.001*
Total encounters, n	84,595	62,580			
Daily rate, mean	308.7	229.2			

IRR = incidence rate ratio; CI = confidence interval; *Significant p-value

Among PED encounters with valid CTAS coding, low-acuity visits (CTAS 4–5) accounted for 62,856 of 77,498 encounters (81.1%) in the pre-implementation period and 30,496 of 60,737 encounters (50.2%) in the post-implementation period. In the post-implementation period, the PUCC managed 24,962 triaged encounters, of which 24,719 (99.0%) were classified as CTAS 4–5. When PED and PUCC activity were considered together during the post-implementation period, 55,215 of 85,699 encounters (64.4%) were low acuity. These findings indicate redistribution of low-acuity activity across care settings rather than a direct reduction in underlying low-acuity demand. The CTAS distribution across PED pre-implementation, PED post-implementation, and PUCC post-implementation encounters is shown in Table 4; Fig. 1.

**Table 4:** CTAS acuity distribution across PED pre-implementation, PED post-implementation, and PUCC post-implementation encounters

Period	CTAS 1–3, *n* (%)	CTAS 4–5, *n* (%)	Total valid CTAS 1–5, *n*
PED Pre	14,642 (18.9%)	62,856 (81.1%)	77,498
PED Post	30,241 (49.8%)	30,496 (50.2%)	60,737
PUCC Post	243 (1.0%)	24,719 (99.0%)	24,962
PED + PUCC Post	30,484 (35.6%)	55,215 (64.4%)	85,699

CTAS = Canadian Triage and Acuity Scale; PED = pediatric emergency department; PUCC = Pediatric Urgent Care Clinic. Low acuity was defined a priori as CTAS levels 4–5. Percentages were calculated among encounters with valid CTAS 1–5 coding

**Fig. 1:**
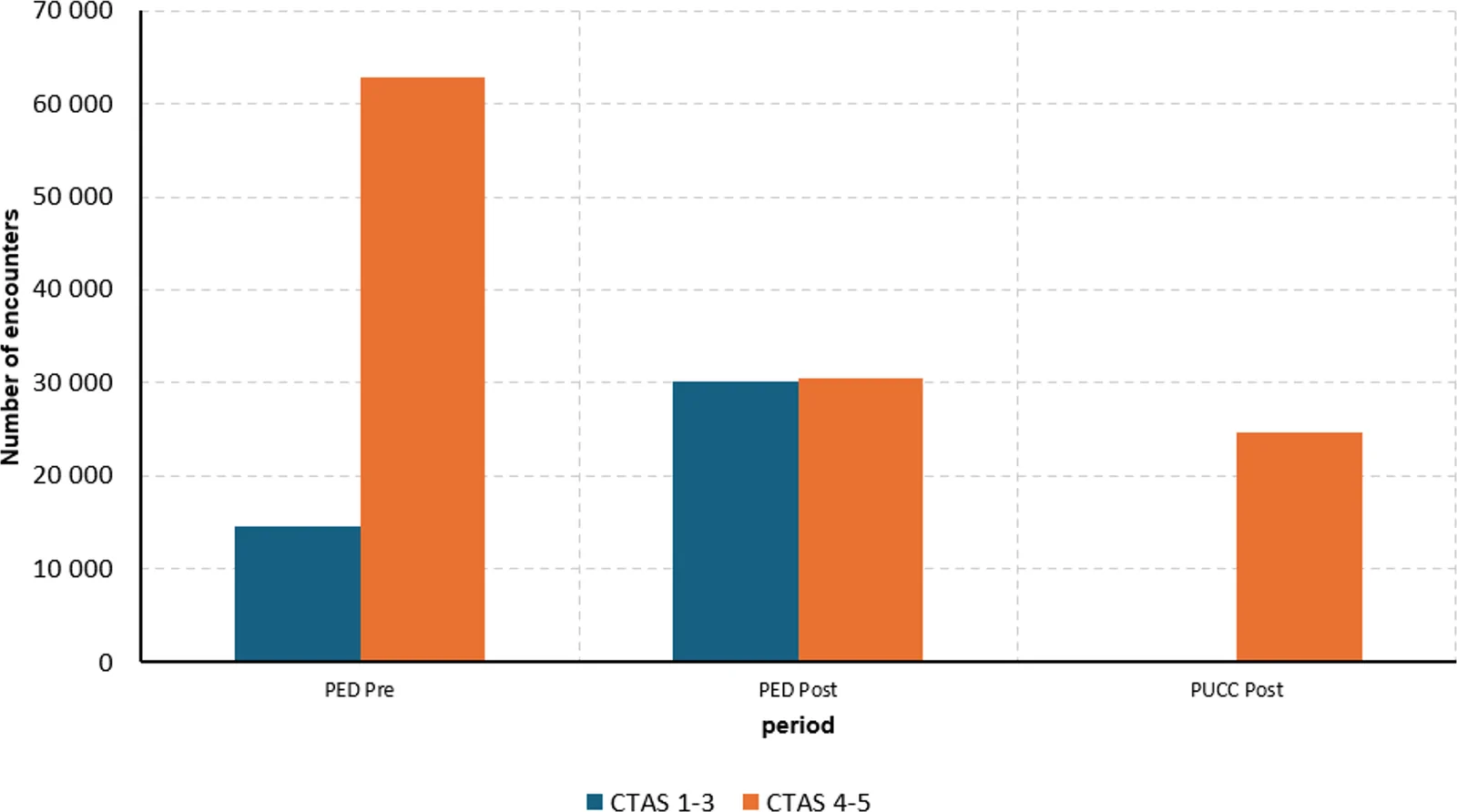
Higher- and low-acuity encounter distribution across PED Pre, PED Post, and PUCC Post periods

Figure 2 shows daily PED encounter volumes during the pre- and post-implementation periods. Patient-flow metrics for PED encounters are summarized in Table 5. For door-to-doctor time, 59,708 encounters were available for analysis in the pre-implementation period and 53,673 in the post-implementation period. The median door-to-doctor time was 37 min (IQR 22–63) pre-implementation and 39 min (IQR 22–65) post-implementation. The corresponding 5% trimmed means were 43.58 and 45.03 min, respectively. For length of stay, 51,417 encounters were available for analysis in the pre-implementation period and 48,141 in the post-implementation period. The median length of stay was 62 min (IQR 34–112) pre-implementation and 76 min (IQR 43–131) post-implementation. The corresponding 5% trimmed means were 78.65 and 92.74 min, respectively. The distributional comparisons for both time-based outcomes were statistically significant (*p* < 0.001).

**Fig. 2:**
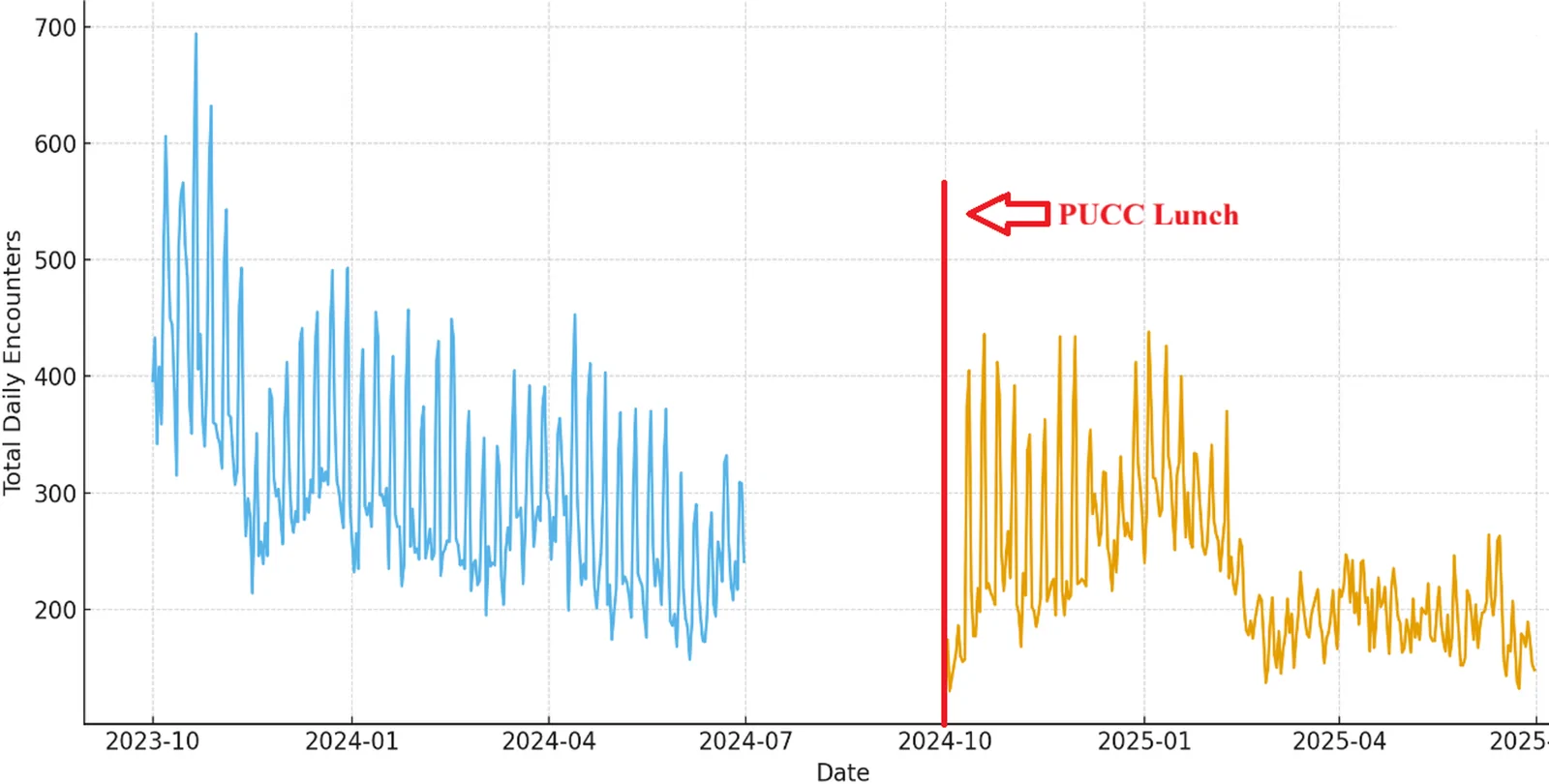
Daily pediatric emergency department encounter volumes before and after PUCC implementation. Blue lines show total daily PED encounters during the pre-implementation window (1 Oct 2023–30 Jun 2024), orange lines show encounters during the post-implementation window (1 Oct 2024–30 Jun 2025), and the vertical red line marks the date of PUCC launch

**Table 5:** Patient-flow indicators before and after PUCC implementation

Indicator		PED Pre	PED Post	*p*-value
Door-to-doctor time (min)	N	59,708	53,673	< 0.001*
	Median, IQR	37.0 (IQR 22.0–63.0)	39.0 (IQR 22.0–65.0)	
	Trimmed mean	43.58	45.03	
Length of stay (min)	N	51,417	48,141	< 0.001*
	Median, IQR	62.0 (IQR 34.0–112.0)	76.0 (IQR 43.0–131.0)	
	Trimmed mean	78.65	92.74	

Values shown as *N*, median (IQR), and 5% trimmed mean. *Significant p-values from Mann–Whitney U test comparing distributions between periods

## Discussion

In this pre–post observational study, implementation of a Pediatric Urgent Care Clinic was associated with lower PED visit volume, a lower proportion of low-acuity PED encounters, and largely stable door-to-doctor times, while PED length of stay increased in the post-implementation period. Taken together, these findings suggest that the PUCC may have contributed to redistribution of lower-acuity pediatric encounters within the hospital system. However, given the before–after design, these associations should be interpreted cautiously and should not be taken as evidence of a causal effect.

The reduction in PED low-acuity activity observed after PUCC implementation is directionally consistent with prior literature showing that urgent care services, retail clinics, and fast-track pathways can absorb lower-acuity demand and reduce pressure on emergency departments [19–23]. In our study, the PUCC managed a predominantly CTAS 4–5 case mix, while the proportion of CTAS 4–5 encounters within the PED decreased during the post-implementation period. These findings are compatible with a redistribution of lower-acuity presentations, although unmeasured operational or behavioral factors may also have contributed.

Door-to-doctor time remained similar across periods, whereas PED length of stay was longer in the post-implementation period. This pattern is also compatible with previous reports indicating that front-end demand management does not necessarily improve overall emergency department throughput when downstream constraints remain unchanged [21, 24–26]. Published evidence suggests that overall LOS is often influenced more strongly by hospital-wide factors, including inpatient bed availability, boarding, consultation delays, and discharge processes, than by arrival volume alone [27–28]. In this context, the stable door-to-doctor interval observed in our study may indicate preserved initial assessment performance, while the longer LOS may reflect pressures beyond the scope of the urgent care intervention itself.

Because the present analysis used CTAS to define acuity, it is relevant that PaedCTAS has demonstrated validity and inter-rater reliability across multiple settings and correlates with admission, resource utilization, intensive care use, and ED length of stay [28–30]. This supports the use of CTAS 4–5 encounters as a pragmatic marker of lower-acuity activity in service-level evaluations. In addition, prior reports from Saudi Arabia support the feasibility of CTAS implementation in the regional context [31].

Our findings should also be interpreted within the local health system context. Prior national and single-center studies from Saudi Arabia have reported PED length-of-stay values in a similar range and have shown that LOS may vary according to triage category, admission status, hospital type, and shift timing [32]. These factors may have influenced post-implementation LOS independently of PUCC availability. In addition, the opening of a new urgent care service may not only redirect existing demand but may also generate new demand by attracting families who might otherwise have delayed care or sought care in another setting. This possibility of induced demand should be considered when interpreting post-implementation utilization patterns.

Several additional sources of potential bias should be acknowledged. First, because this was a real-world observational evaluation, patients were not randomly allocated between care settings, and unmeasured differences in case mix or operational workflow may have influenced the results. Second, the present dataset did not include all service-level variables that would be useful for a fuller assessment of the intervention, such as transfer from PUCC to PED, downstream safety outcomes, revisit rates, or complications after urgent care evaluation. These outcomes are important for determining whether redistribution of patient flow was accompanied by preserved quality and safety. Third, if financial, administrative, or access differences existed between the PUCC and PED, these factors may also have influenced care-seeking behavior; although both services operated within the same institutional framework, the potential effect of such factors should be considered in similar future evaluations.

This study has several strengths. It included a large operational dataset, prespecified data-cleaning rules, and standardized acuity-based outcome definitions. The use of matched calendar periods reduced, although did not eliminate, the influence of seasonality. At the same time, several limitations are important. The before–after design remains susceptible to secular trends, contemporaneous operational changes, and residual confounding. Time-stamp completeness improved between periods, which may have affected some comparisons. In addition, disposition taxonomies differed across study periods; for this reason, admission-related comparisons were not considered robust enough to serve as a principal comparative outcome. Future studies using interrupted time-series methods, adjustment for shift and weekday patterns, and inclusion of boarding, safety, and revisit metrics would provide a stronger basis for causal inference.

Overall, the present findings suggest that a pediatric urgent care model may be associated with redistribution of lower-acuity encounters away from the PED while preserving initial physician assessment times. However, improvement in overall throughput is unlikely to depend on front-end redesign alone. Hospital-wide flow measures, including strategies addressing inpatient capacity and discharge processes, may still be required to improve total visit length [21, 33]. Further evaluation in Saudi Arabia and similar health systems should examine not only utilization and acuity patterns, but also safety, revisit behavior, transfer between care settings, and patient-centered outcomes.

## Conclusion

Implementation of a Pediatric Urgent Care Clinic at our institution was associated with lower PED visit volume, a reduced proportion of low-acuity PED encounters, and largely unchanged door-to-doctor times, while PED length of stay was longer in the post-implementation period. These findings suggest that a PUCC model may help redistribute lower-acuity pediatric encounters within a hospital-based urgent and emergency care system. However, because this was a pre–post observational study, the findings should be interpreted cautiously and should not be taken to imply causality. Further studies incorporating safety outcomes, revisit rates, transfer between care settings, and stronger adjustment for operational confounding are needed to better define the role of pediatric urgent care services in optimizing emergency department utilization.

## Supplementary Information

Below is the link to the electronic supplementary material.

Supplementary Material 1

## Data Availability

The datasets supporting the conclusions of this article are available from the corresponding author upon reasonable request. The data are not publicly available due to institutional data governance policies and restrictions related to patient confidentiality.
