# Supplementary material for: Impact of pediatric urgent care clinics on emergency department utilization and patient flow: a pre–post cohort study in Saudi Arabia
Source: BMC Emerg Med. 2026 Jul 8;26:261. doi: 10.1186/s12873-026-01670-0 (PMC13637253; doi:10.1186/s12873-026-01670-0)
Supplement: Supplementary file 1 [file 12873_2026_1670_MOESM1_ESM.docx]

**Supplementary Table 1. Institutional triage and PUCC eligibility pathway**

| **Component** | **PED pathway** | **PUCC pathway** |
| --- | --- | --- |
| Service role | 24-hour emergency service for all acuity levels, including unstable patients and those requiring emergency-level resources | Defined-hours urgent care pathway for clinically stable lower-acuity pediatric presentations |
| Target acuity | CTAS 1–2, unstable CTAS 3, and any child requiring emergency evaluation, monitoring, resuscitation, or immediate specialist input | CTAS 4–5 and selected stable CTAS 3 presentations not requiring emergency-level resources |
| Eligible clinical profile | Children with potentially serious, unstable, or high-risk presentations | Clinically stable children with lower-acuity acute complaints |
| Examples of cases | Respiratory distress, shock, altered consciousness, severe dehydration, major trauma, suspected sepsis, seizures, or other unstable/high-risk presentations | Fever without instability, upper respiratory symptoms, mild gastrointestinal illness, minor injuries, and other stable low-acuity complaints |
| Exclusion from PUCC |  | Clinical instability, need for resuscitation, advanced monitoring, emergency-level diagnostics or treatment, urgent specialist intervention, or deterioration during assessment |
| Escalation process | Patients remain in the PED or are transferred to higher-level care if needed | Patients are redirected or transferred to the PED if the condition exceeds PUCC capacity or deterioration occurs |
| Study classification | Encounters classified as PED using site/location identifiers in the EHR | Encounters classified as PUCC using site/location identifiers in the EHR |

PED = pediatric emergency department; PUCC = Pediatric Urgent Care Clinic; CTAS = Canadian Triage and Acuity Scale; EHR = electronic health record.
